# Restricted Presence of POU6F2 in Human Corneal Endothelial Cells Uncovered by Extension of the Promoter-level Expression Atlas

**DOI:** 10.1016/j.ebiom.2017.10.024

**Published:** 2017-11-04

**Authors:** Masahito Yoshihara, Susumu Hara, Motokazu Tsujikawa, Satoshi Kawasaki, Yoshihide Hayashizaki, Masayoshi Itoh, Hideya Kawaji, Kohji Nishida

**Affiliations:** a Department of Ophthalmology, Osaka University Graduate School of Medicine, Suita, Osaka 565-0871, Japan; b Division of Genomic Technologies, RIKEN Center for Life Science Technologies, Yokohama, Kanagawa 230-0045, Japan; c Department of Stem Cells and Applied Medicine, Osaka University Graduate School of Medicine, Suita, Osaka 565-0871, Japan; d Department of Visual Regenerative Medicine, Osaka University Graduate School of Medicine, Suita, Osaka 565-0871, Japan; e Department of Ocular Immunology and Regenerative Medicine, Osaka University Graduate School of Medicine, Suita, Osaka 565-0871, Japan; f RIKEN Preventive Medicine and Diagnosis Innovation Program, Wako, Saitama 351-0198, Japan; g Preventive Medicine and Applied Genomics Unit, RIKEN Advanced Center for Computing and Communication, Yokohama, Kanagawa 230-0045, Japan

**Keywords:** Transcriptome, Corneal endothelial cells, Cell-type specific marker, Transcription factor, Gene expression, Regenerative medicine, CEC, corneal endothelial cell, CE tissue, corneal endothelial tissue, HCEPs, human corneal endothelial progenitors, dHCEPs, differentiated human corneal endothelial progenitors, CAGE, cap analysis of gene expression, FANTOM, Functional Annotation of Mammalian Genome, tpm, tags per million

## Abstract

Corneal endothelial cells (CECs) are essential for maintaining the clarity of the cornea. Because CECs have limited proliferative ability, interest is growing in their potentially therapeutic regeneration from pluripotent stem cells. However, the molecular mechanisms of human CEC differentiation remain largely unknown. To determine the key regulators of CEC characteristics, here we generated a comprehensive promoter-level expression profile of human CECs, using cap analysis of gene expression (CAGE) with a single molecule sequencer. Integration with the FANTOM5 promoter-level expression atlas, which includes transcriptome profiles of various human tissues and cells, enabled us to identify 45 promoters at 28 gene loci that are specifically expressed in CECs. We further discovered that the expression of transcription factor POU class 6 homeobox 2 (POU6F2) is restricted to CECs, and upregulated during human CEC differentiation, suggesting that POU6F2 is pivotal to terminal differentiation of CECs. These CEC-specific promoters would be useful for the assessment of fully differentiated CECs derived from pluripotent stem cells. These findings promote the development of corneal regenerative medicine.

## Highlights

• We comprehensively profiled promoter-level expression of human corneal endothelial cells.
• Integrative transcriptome analysis identified 28 corneal endothelial cell-specific marker genes.
• POU6F2 expression is restricted to corneal endothelial cells, and upregulated during differentiation.

Corneal endothelial cells (CECs) are essential for maintaining corneal transparency. Owing to the high prevalence of corneal endothelial disorders, CECs are important targets in regenerative medicine. However, it has been difficult to evaluate the final CEC products owing to the lack of appropriate CEC-specific markers. In this study, we identified 28 CEC-specific marker genes by integrative transcriptome analysis. One gene of particular interest, POU6F2, is expressed almost exclusively in CECs, and upregulated during differentiation. These markers would be useful for the assessment of CECs derived from pluripotent stem cells, and this study will facilitate the translation of corneal regenerative medicine.

## 1 Introduction

Cornea is a transparent, avascular tissue located at the front of the eye. Corneal endothelium is the innermost monolayer of the cornea attached to Descemet's membrane. Corneal endothelial cells (CECs) play a crucial role in the maintenance of corneal transparency, by controlling the movement of ions and water between the corneal stroma and the anterior chamber (Hodson and Miller, 1976, Maurice, 1972). Because human CECs have limited proliferative ability (Joyce et al., 1996), significant loss of CECs due to disease or trauma can cause corneal edema, corneal opacification, and, consequently, impaired vision. At present, allogeneic corneal transplantation is the most effective way to treat corneal endothelial dysfunction. However, this procedure is limited by a global scarcity of healthy donors (Shimazaki et al., 2004).

A number of technologies have been developed to use cultured CECs as an alternative to donor corneal endothelium (Engelmann et al., 1988, Joyce and Zhu, 2004, Mimura et al., 2013, Proulx and Brunette, 2012, Sumide et al., 2006). However, it is extremely difficult to culture human CECs for long periods (Peh et al., 2011). This difficulty is encountered because cultured CECs easily lose typical CEC characteristics, by switching their phenotype from endothelial to fibroblastic (Okumura et al., 2013) in a process referred to as endothelial-to-mesenchymal transition (Roy et al., 2015), which limits the use of cultured CECs for the treatment of corneal endothelial disorders. To solve this problem, recent tissue engineering studies have focused on the development of alternative CECs from other cell types, such as the iris (Kikuchi et al., 2011) and corneal stroma (Hatou et al., 2013). More recent studies successfully induced human embryonic stem cells to develop into CEC-like cells (Song et al., 2016, Zhang et al., 2014). Given the recent rapid progress in the field of stem cell research, a method to produce CECs from induced pluripotent stem cells is likely to be developed in the near future.

Since pluripotent stem cells can differentiate into various cell types, CEC-specific markers are necessary for the evaluation of the final products. Moreover, to reproduce the developmental process of human CECs *in vitro*, it is also essential to understand the molecular dynamics of human CEC differentiation. Several studies demonstrated that the neural crest-derived periocular mesenchyme gives rise to corneal endothelium and stroma, trabecular meshwork, iris, ciliary body, and sclera (Cvekl and Tamm, 2004, Gage et al., 2005, Williams and Bohnsack, 2015). These studies revealed that PITX2 is required for the differentiation of the neural crest-derived periocular mesenchyme during early ocular development in mice (Gage et al., 2005). Mutations in *PITX2* are associated with Axenfeld-Rieger syndrome, which is characterized by dysgenesis of anterior segment, including corneal endothelium (Kozlowski and Walter, 2000, Lines et al., 2002). These observations indicate that PITX2 plays a crucial role in the development of the human neural crest-derived periocular mesenchyme. However, key regulators of human CEC lineage commitment from periocular mesenchyme remain to be elucidated. We previously isolated human corneal endothelial progenitors (HCEPs) from CECs, and successfully converted these HCEPs into differentiated HCEPs (dHCEPs) that had pump function similar to that of CECs (Hara et al., 2014).

Pursuing a comprehensive molecular understanding of human CECs and their differentiation process, here we explored transcriptome characteristics of human CECs, including HCEPs and dHCEPs, using cap analysis of gene expression (CAGE), which enabled us to monitor promoter activities at the genome-wide level (Shiraki et al., 2003). First, we identified specific markers of CECs by referring to the Functional Annotation of Mammalian Genome 5 (FANTOM5) expression atlas, which catalogs promoter activities in a wide variety of human tissue and cell samples (Forrest et al., 2014). Next, we identified transcription factors that are specifically expressed in CECs, which might control the cell fate and lineage commitment of CECs. Finally, we analyzed transcriptional dynamics during human CEC differentiation, and found that the majority of CEC-specific promoters are upregulated during differentiation. These findings may facilitate selective differentiation of CECs *in vitro*, and thereby accelerate the development of corneal regenerative medicine.

## 2 Materials and Methods

### 2.1 Preparation of Human Corneal Endothelial Samples for CAGE Analysis

The use of all human samples in this study adhered to the tenets of the Declaration of Helsinki. Research-grade corneoscleral rims and whole eye globes from cadaver human donors were obtained from SightLife (Seattle, WA, USA). Informed consent for eye donation to research was obtained from the next of kin of all deceased donors by SightLife.

#### 2.1.1 Preparation of Human Corneal Endothelial Tissues

To obtain the freshest possible corneal endothelial samples, we recovered 36 corneal endothelial (CE) tissues within a few days following death (22 ± 13 h), and before shipping (Fig. S1a). Descemet's membranes with the corneal endothelial monolayer were carefully dissected from corneoscleral rims, using sterile surgical forceps, as described previously (Yoshihara et al., 2015). The stripped Descemet's membranes with endothelium were immediately transferred into RNAlater RNA Stabilization Reagent (QIAGEN Inc., Valencia, CA, USA). Among these tissue samples, three from healthy donors with high RNA quality were analyzed by CAGE.

#### 2.1.2 Preparation of Cultured CECs, HCEPs, and dHCEPs

To cultivate human CECs, Descemet's membranes with their endothelium were treated with enzyme-containing cell detachment medium (Accutase; Life Technologies, Grand Island, NY, USA) at 37 °C for 30 min, and seeded onto culture dishes coated with 0.1 μg/cm^2^ laminin-511E8 (Wako Pure Chemical Industries, Osaka, Japan) in Dulbecco's modified Eagle's medium (DMEM; Life Technologies), supplemented with 10% fetal bovine serum (FBS; Japan Bio Serum, Hiroshima, Japan) and 2 ng/mL basic fibroblast growth factor (bFGF; Wako Pure Chemical Industrials). CECs at the proliferation stage were collected and subcultured when they reached 70% confluence, and collected again when they reached 100% confluence.

HCEPs and dHCEPs were obtained according to previously described procedures (Hara et al., 2014). Briefly, the Descemet's membranes were stripped from the corneas in DMEM, and treated with Accutase at 37 °C for 30 min. The detached CECs were seeded at a density of 100–300 cells/cm^2^ onto culture plates coated with 0.1 μg/cm^2^ laminin-511E8. The medium was composed of DMEM/Nutrient Mixture F-12 (DMEM/F12; Life Technologies) containing 20% Knockout Serum Replacement (KSR; Life Technologies), 2 mM l-glutamine (Life Technologies), 1% non-essential amino acids (Life Technologies), 100 μM 2-mercaptoethanol (Life Technologies), 50 U/mL penicillin G, 50 μg/mL streptomycin (Life Technologies), and 4 ng/mL bFGF. The culture medium was changed every 2–3 days. When the cells reached 70% confluence, they were harvested with Accutase and passaged at ratios of 1:2–1:5. HCEPs were differentiated into mature CECs (*i.e.*, differentiated HCEPs: dHCEPs) on dishes coated with FNC coating mix (AthenaES, Baltimore, MD, USA). The differentiation medium consisted of DMEM supplemented with 10% FBS, 50 U/mL penicillin G, and 50 μg/mL streptomycin. The cells were cultured at 37 °C in an atmosphere of 95% air and 5% CO_2_ for 28 days.

#### 2.1.3 RNA Preparation From CEC Samples

Total RNA was extracted from tissues or cells, using an miRNeasy Mini Kit (QIAGEN Inc.), according to the manufacturer's instructions. The quantity and quality of the extracted RNA was determined using a NanoDrop spectrophotometer (Thermo Fisher Scientific, Waltham, MA, USA) and an Agilent BioAnalyzer 2100 (Agilent Technologies, Santa Clara, CA, USA). The RNA integrity number (RIN) of each sample is shown in Table S1.

### 2.2 CAGE Analysis and Data Processing

#### 2.2.1 CAGE Library Preparation

CAGE libraries were prepared from total RNA, as previously described (Kanamori-Katayama et al., 2011), using SuperScript III Reverse Transcriptase (Life Technologies, Carlsbad, CA, USA) for reverse transcription, NaIO_4_ for diol oxidation, biotin hydrazide (Vector Laboratories, Burlingame, CA, USA) for biotinylation, RNase I (Promega, Madison, WI, USA) for single-strand RNA digestion, streptavidin-coated magnetic beads (Dynabeads M-270 Streptavidin; Life Technologies) for biotinylated RNA/cDNA recovery, and an Agencourt AMPure XP Kit (Beckman Coulter, Brea, CA, USA) for purification and buffer exchange. After polyA tailing reaction using terminal transferase and dATP, cDNAs were blocked with ddATP. The resulting CAGE libraries were loaded on two lanes of a HeliScope single molecule sequencer (Helicos Biosciences, Cambridge, MA, USA). An overview of the sequencing data is presented in Table S1. All CAGE sequence data analyzed in this study were deposited to the DNA Data Bank of Japan (DDBJ) Sequence Read Archive (http://trace.ddbj.nig.ac.jp/dra/index_e.html) under accession number DRA005836.

#### 2.2.2 Annotation of Promoters and Differential Expression Analysis

After base calling, raw reads containing base-order addition artifacts, and other low-quality reads, were removed using an SMS filter program supplied by Helicos. In addition, reads shorter than 20 nucleotides and longer than 70 nucleotides were removed. These filtered reads were mapped to the human genome sequence (hg19), using Delve (Djebali et al., 2012) and the MOIRAI pipeline platform (Hasegawa et al., 2014). Mapped reads (tags) were counted with respect to the robust peaks identified in the FANTOM5, which was used as a reference for promoter regions (Forrest et al., 2014). On the basis of the total number of tags, CAGE peaks associated with a single gene were labeled as p1, p2, and so forth. For example, p1@PITX2 corresponds to one of the alternative promoters of *PITX2,* which has the highest tag counts in the FANTOM5. In this study, we regarded p1–p3 as major promoters. Raw tag counts generated from duplicated sequencing were merged, and subsequently normalized against total tags per sample, by the relative log expression (RLE) method (Anders and Huber, 2010). For the identification of CEC-specific promoters, the FANTOM5 expression tables were downloaded from http://fantom.gsc.riken.jp/5/. CAGE tag count data from human tissues or primary cells were combined with those of CE tissues or cultured CECs, and differential expression was analyzed using the Bioconductor package edgeR (version 3.10.2) (Robinson et al., 2010). Promoters that were differentially expressed between HCEPs and dHCEPs were defined as having a mean fold change > 2 and Benjamini-Hochberg (BH)-adjusted *P* < 0.01 between pairs of donors. Gene ontology (GO) enrichment analysis of the differentially expressed genes was performed using the DAVID web tool (http://david.abcc.ncifcrf.gov/).

#### 2.2.3 RNA-seq Data Processing

RNA-seq data on expression profiles of three adult CECs and two fetal CECs were downloaded from the Gene Expression Omnibus (GEO) database (Edgar et al., 2002), under the accession number GSE41616 (Chen et al., 2013). One adult sample (GSM1020213) was excluded from the analysis because corneal epithelial cells were considered to be contaminated, as we previously reported (Yoshihara et al., 2015). After a quality check using FastQC (http://www.bioinformatics.babraham.ac.uk/projects/fastqc/), the processed reads were aligned to hg19, using Tophat (version 1.4.1) (Trapnell et al., 2012). Total read counts mapped to each gene were quantified using HTSeq v0.5.4p3 (Anders et al., 2015). Genes with < 10 reads in any sample were removed. The read counts were normalized by the RLE method, and differential expression was analyzed using the Bioconductor package edgeR, based on a mean fold change > 2 and BH-adjusted *P <* 0.01.

### 2.3 Experimental Validation

#### 2.3.1 RNA Preparation From Human Ocular and Non-ocular Tissues

RNA samples from ocular tissues were prepared as previously described (Yoshihara et al., 2015). Briefly, each tissue was carefully isolated from four whole globes of two donors, using sterile surgical forceps. Central cornea and limbus were divided with an 8.0-mm diameter trephine, and treated with Dispase I (Godo Shusei, Tokyo, Japan) overnight at 4 °C to separate corneal epithelium and limbal epithelium from stroma. All isolated tissues were rapidly transferred into Isogen RNA extraction reagent (Nippon Gene, Tokyo, Japan), and total RNA was extracted using an Isogen RNA extraction kit.

We purchased non-ocular tissue RNA samples as follows: Human total RNA master panel II #636643 (Clontech, Mountain View, CA, USA); Human Kidney Total RNA (#AM7976; Ambion, Austin, TX, USA); and Human Pancreas Total RNA (#AM7954; Ambion).

#### 2.3.2 Quantitative Reverse Transcription (qRT)-PCR

cDNAs were synthesized using a SuperScript III first-strand synthesis system for qRT-PCR (Life Technologies), according to the manufacturer's protocol. TaqMan probe mixtures and primers were purchased from Life Technologies (Table S2). Quantitative PCR was carried out using the QuantStudio K12 Flex Real-Time PCR System (Life Technologies). Expression values were normalized to those of glyceraldehyde 3-phosphate dehydrogenase (*GAPDH*) gene which we used as an internal control.

#### 2.3.3 Immunofluorescence Staining

The corneal tissues were fixed with 4% paraformaldehyde at 4 °C for 30 min. Non-specific absorption was blocked in the samples using a 5% solution of normal donkey serum in Tris-buffered saline and permeabilized with 0.3% Triton X-100. The tissues were next incubated at 4 °C, for 2 days, with primary antibodies against the POU class 6 homeobox 2 (POU6F2) protein (1:100; RRID: AB_11149941; Santa Cruz Biotechnology, Santa Cruz, CA, USA) and ZO-1 (1:100; Cat #13663; Cell Signaling Technology, Danvers, MA, USA) in Tris-buffered saline, containing 1% normal donkey serum and 0.3% Triton-X 100. The tissues were incubated with Alexa Fluor-568-conjugated anti-mouse IgG and Alexa Flour-647-conjugated anti-rabbit IgG (RRID: AB_2534013 and RRID: AB_2536183; Life Technologies) at room temperature for 2 h. They were then counterstained with 5 μg/mL Hoechst 33342 (Life Technologies). For secondary staining, the tissues were incubated with Alexa-488-conjugated antibodies against p75 neurotrophin receptor (p75NTR) (1:100; RRID: AB_10972736; Becton, Dickinson and Company, Franklin Lakes, NJ, USA) at 4 °C overnight and mounted with PermaFlour (Thermo Fisher Scientific). The specimens were observed under a confocal fluorescence microscope (LSM710; Carl Zeiss, Jena, Germany).

#### 2.3.4 Western Blotting

HCEPs and dHCEPs were harvested by scraping, and lysed in RIPA buffer supplemented with a complete protease inhibitor cocktail (Roche, Penzberg, Germany). Total lysate protein (10 μg) was electrophoresed on SDS-polyacrylamide gels before being transferred to polyvinylidene difluoride membranes. The membrane was treated with 5% skim milk containing TBS-T (Takara Bio, Shiga, Japan), and probed with an anti-POU6F2 antibody (1:1000; RRID: AB_10711285; Abcam, Cambridge, USA) and an anti-GAPDH antibody (1:1000; RRID: AB_627679; Santa Cruz) at 4 °C overnight. Next, the membrane was washed three times, and probed with an HRP-conjugated anti-mouse IgG antibody at room temperature for 1 h. The membrane was then treated using an ECL Prime Western Blot Detection Kit (GE Healthcare, Little Chalfont, Buckinghamshire, UK), and images were generated using the ChemiDoc XRS gel imaging system (Bio-Rad, Hercules, CA, USA).

## 3 Results

### 3.1 Transcriptome Profiling of Different Preparations of Human CECs

**Fig. 1:**
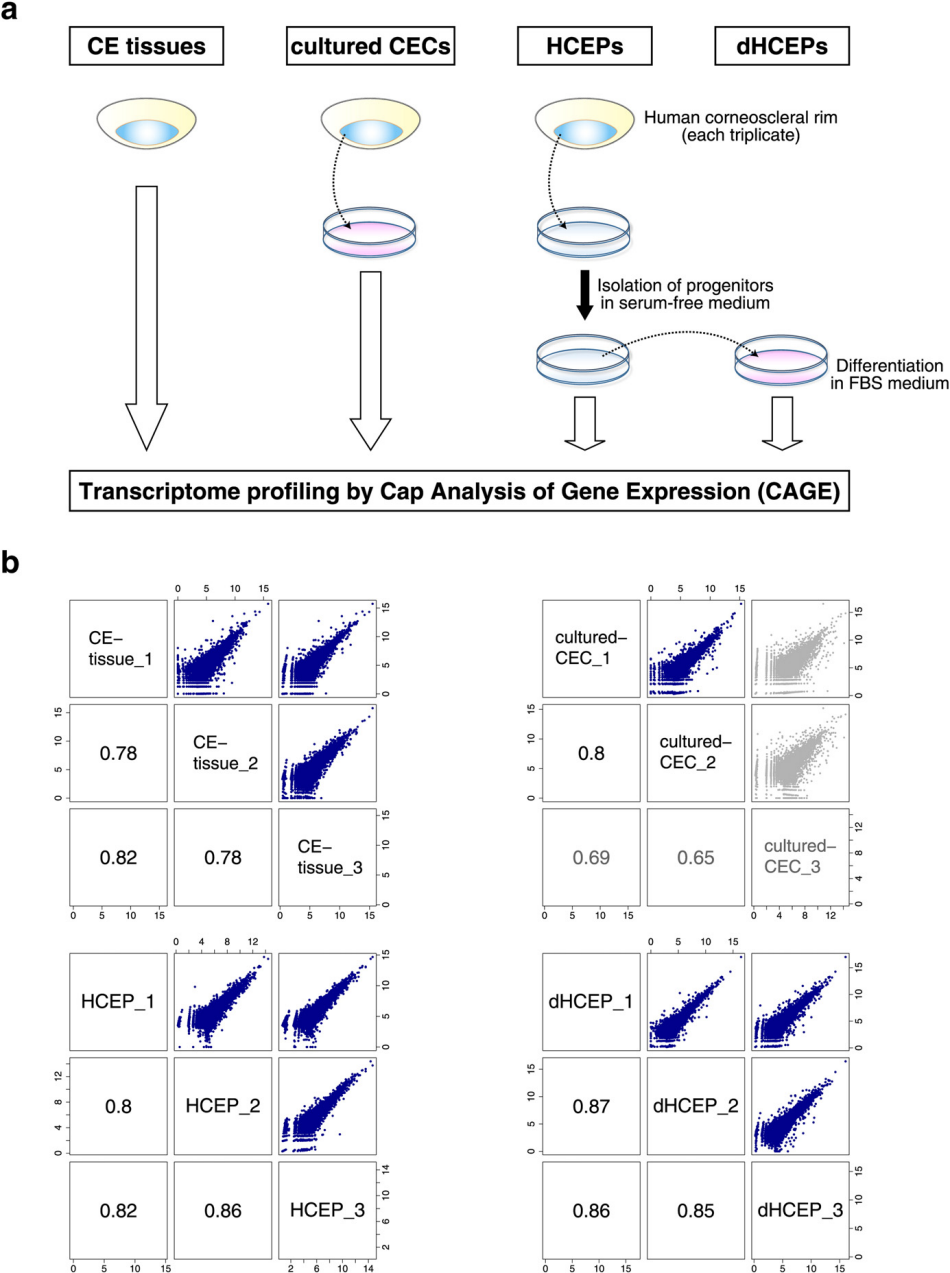
Study design and quality check. (a) Study design. Corneal endothelia were dissected from corneoscleral rims derived from three donors for each type of sample: corneal endothelial (CE) tissues, cultured corneal endothelial cells (CECs), and corneal endothelial progenitor cells (HCEPs). For CE tissues, RNA was extracted directly from dissected corneal endothelium. For cultured CECs, RNA was extracted from CECs after *ex vivo* expansion. HCEPs were isolated in serum-free culture media (shown in blue) and differentiated into mature CECs (dHCEPs) by being cultured in differentiation media containing fetal bovine serum (shown in red). RNA was extracted from both HCEPs and dHCEPs. Each RNA sample was processed and analyzed by CAGE. (For interpretation of the references to color in this figure legend, the reader is referred to the web version of this article.) (b) Correlation analysis of promoter activities between each triplicate. Each number represents the Spearman's rank correlation coefficient. Numbers and dots shown in gray indicate low correlation of “cultured-CEC_3” expression profiles with those of the other two cultured CEC samples. The x- and y-axes represent log_2_-scaled expression values (tpm) for each promoter.

To explore human CECs and their differentiation process at the molecular level, we made four human CEC preparations: CE tissues, cultured CECs, corneal endothelial progenitor cells isolated from cultured CECs (HCEPs), and *in vitro*-differentiated HCEPs (dHCEPs) (Fig. 1a). Given the number of human CECs *in vivo* (~ 4 × 10^5^ cells (Kitazawa et al., 2016)), the amounts of total RNA previously extracted from CE tissue have been extremely low (~ 0.2 μg). This paucity might be because RNA is not fully maintained during shipping; it usually takes ~ 1 week to obtain corneal tissues after excision (Hara et al., 2014). To minimize the loss of RNA after tissue excision, within a few days following death, and prior to shipping, we collected CE tissues from cadavers and transferred them into an RNA preservation reagent. As a result, the amount of total RNA that we extracted from these fresh CE tissues was relatively high (1.0 ± 0.4 μg) (Fig. S1a).

With sufficient amounts of high-quality RNA extracted from CECs, we generated a comprehensive promoter-level expression profile of these CEC preparations by CAGE using a HeliScope single molecule sequencer, following the protocols used in the FANTOM5 (Forrest et al., 2014). For each CEC preparation, biological samples were processed and analyzed in triplicate (Table S1). HCEP and dHCEP pairs were derived from three identical donors (Fig. 1a). To assess the validity of our approach, we initially performed a correlation analysis of promoter activities between each triplicate. Although most of the pairs showed high correlation (ρ > 0.77, Spearman's rank correlation coefficient) (Fig. 1b), the third replicate of the cultured CEC (“cultured-CEC_3”) sample showed an expression pattern different from those of the other two cultured CEC samples (Fig. 1b, gray). Furthermore, well-known CEC markers, such as *SLC4A11* and *COL8A2* (Chng et al., 2013), were expressed at very low levels in this sample, relative to the levels seen in the other CE tissue and cultured CEC samples (Fig. S1b). These observations suggested that “cultured-CEC_3” cells lost CEC characteristics, and we therefore excluded this set from the following analyses.

### 3.2 Identification of CEC-specific Promoters Across the Human Body

**Fig. 2:**
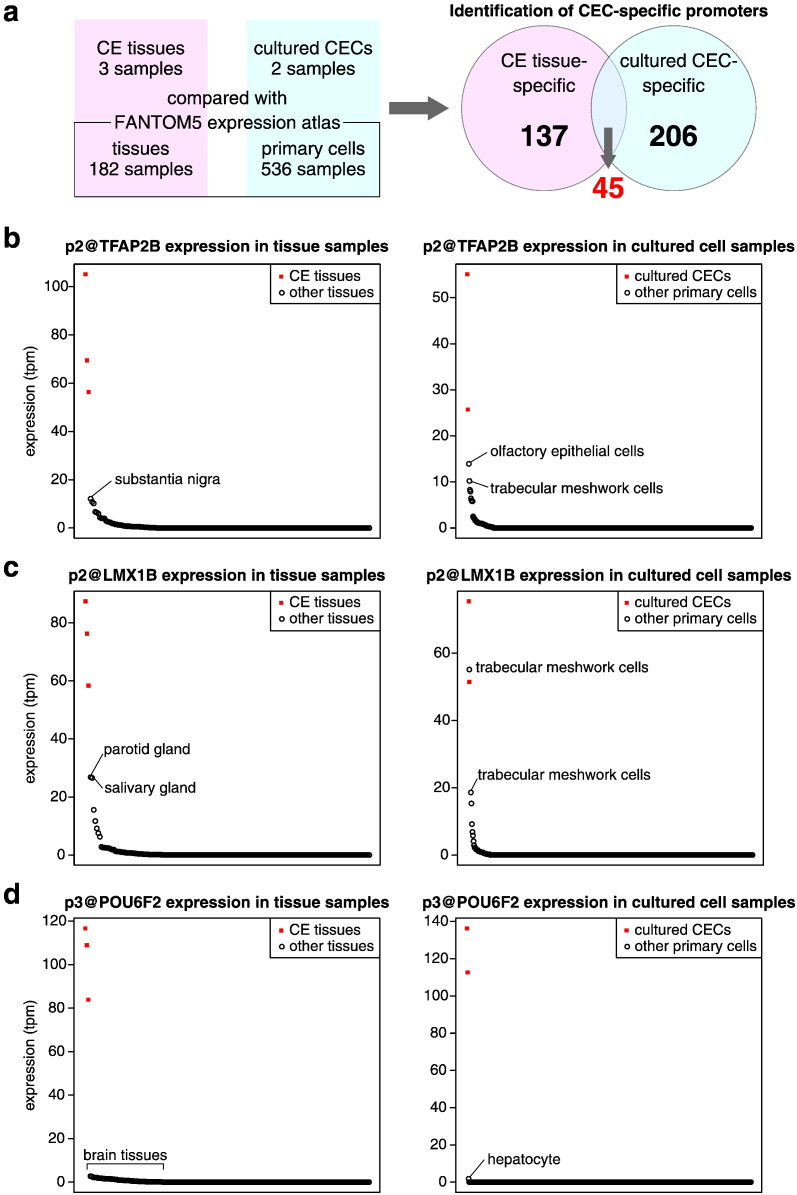
Identification of corneal endothelial cell-specific promoters in the human body. (a) The method and results. CAGE profiling data of three corneal endothelial (CE) tissue samples were compared with those of 182 tissue samples, and CAGE profiling data of two cultured corneal endothelial cell (CEC) samples were compared with those of 536 primary cell samples (left). As a result, 137 CE tissue-specific promoters and 206 cultured CEC-specific promoters were identified. Forty-five promoters were common to both sets of specific promoters (right). (b–d) Expression levels of p2@TFAP2B (b), p2@LMX1B (c), and p3@POU6F2 (d) in CECs and various human tissues or cells. Red squares represent expression levels in CECs, and black circles represent expression levels in other tissues or cells. Samples are sorted from left to right in the order of the expression level of each promoter. The y-axes represent expression levels (tpm).

Taking advantage of CAGE profiling datasets for three CE tissue and two cultured CEC preparations (without “cultured-CEC_3”), we determined their specific promoters. As a resource of gene expression profiles in other types of tissues or cells, we utilized the FANTOM5 promoter atlas, which represents promoter activities in a wide range of human tissue and cell samples quantified by CAGE (Forrest et al., 2014). We compared CAGE profiling data from our three CE tissue preparations with data from 182 tissue samples and, similarly, we compared profiling data from our two cultured CEC preparations with data from 536 primary cell samples (Fig. 2a). It should be noted that CECs are not included in the FANTOM5 atlas, and an eyeball sample in the FANTOM5 atlas was excluded from this analysis, to exclude a potential CEC expression profile from the reference dataset. To determine the promoters that are specifically expressed in CECs in the human body, we set the following criteria: 1) the expression levels in all CEC samples had to be > 10 tags per million (tpm); 2) the mean expression level in other samples was < 3 tpm; 3) the highest expression in other samples was less than the mean expression level in CEC samples; 4) the log_2_ fold change (mean expression level in CEC/other samples) was > 5, and 5) strong statistical significance (adjusted *P*-value < 0.01). These criteria revealed 137 promoters that were specifically expressed in CE tissues (Table S3a), and 206 promoters that were specifically expressed in cultured CECs (Table S3b). Interestingly, 45 promoters of 28 gene loci were specifically expressed in both CE tissues and cultured CECs (Fig. 2a and Table 1). We expect that these 28 CEC-specific genes play important roles in CEC characterization. These genes included the CEC markers *SLC4A11* and *COL8A2* (Chng et al., 2013), which are known for their mutations in corneal endothelial dystrophies (Biswas et al., 2001, Vithana et al., 2006, Vithana et al., 2008) (Table 1 and Fig. S2a, b). They also included miR-184, whose mutation causes EDICT syndrome, which entails corneal endothelial dystrophy, iris hypoplasia, congenital cataract, and stromal thinning (Iliff et al., 2012) (Table 1 and Fig. S2c). Major promoters (p1–p3) of *PITX2*, a key regulator of the neural crest-derived periocular mesenchyme, were also included in this list (Table 1 and Fig. S2d).

Among these 45 promoters at 28 gene loci, we focused on transcription factors in order to identify key regulators that determine the transcriptional network in CECs. In addition to *PITX2*, we identified several other CEC-specific transcription factors. One of the major promoters of *TFAP2B*, p2@TFAP2B, was highly expressed in CECs, and moderately expressed in trabecular meshwork cells (Fig. 2b). p2@LMX1B was highly expressed in CECs, salivary gland, and trabecular meshwork cells (Fig. 2c). Similar to CECs, the latter two tissues originate from the neural crest. This suggests that LMX1B is involved in the differentiation of neural crest-derived cells. Notably, p3@POU6F2 was highly expressed in CECs (~ 100 tpm), whereas its expression in other tissues was limited to only brain tissues at a low level (< 3 tpm). Strikingly, this promoter was not expressed in any other primary cell samples, except for in one hepatocyte sample (1.77 tpm) (Fig. 2d). Although p5@ERG and p6@ZFHX4 were specifically expressed in CECs, main promoters (p1) of *ERG* and *ZFHX4* were highly expressed in other tissues or cells (Table S4). As a result, we considered TFAP2B, LMX1B, and POU6F2 as CEC-specific transcription factors.

To validate the expression of genes encoding these three CEC-specific transcription factors in human tissues, we performed qRT-PCR analysis using CE tissues and 22 other non-ocular tissue samples. This analysis confirmed the CEC-specific expression pattern of these three transcription factors (Fig. S3). In agreement with the CAGE results, *LMX1B* was highly expressed in salivary glands. Our qRT-PCR results also indicated that, among all human tissues tested, *POU6F2* expression was restricted to CECs.

**Table 1:** List of 45 CEC-specific promoters at 28 gene loci.

Gene symbol	Description	Promoters
*ATP6V1G1*	ATPase H + transporting V1 subunit G1	p@chr9:117359938..117359952,+
*C4orf49 (MGARP)*	Mitochondria localized glutamic acid rich protein	p3@C4orf49
*CA12*	Carbonic anhydrase 12	p@chr15:63656527..63656531,−
*COL4A3*	Collagen type IV alpha 3 chain	p3@COL4A3
*COL8A1*	Collagen type VIII alpha 1 chain	p2@COL8A1, p@chr3:99354394..99354405,+
*COL8A2*	Collagen type VIII alpha 2 chain	p2@COL8A2
*DNAJC6*	DnaJ heat shock protein family (Hsp40) member C6	p3@DNAJC6
*ENO1*	Enolase 1	p8@ENO1, p9@ENO1, p12@ENO1
*ENO1P1*	Enolase 1 pseudogene 1	p1@ENO1P1, p@chr1:236647096..236647105,+
*ENST00000354541*		p1@ENST00000354541
*ENST00000357401*		p1@ENST00000357401
***ERG***	ETS transcription factor	p5@ERG
*FGF10*	Fibroblast growth factor 10	p1@FGF10
*FGF7*	Fibroblast growth factor 7	p3@FGF7, p6@FGF7, p10@FGF7
*IGFBP2*	Insulin-like growth factor binding protein 2	p@chr2:217526641..217526672,+
*ITGBL1*	Integrin subunit beta like 1	p3@ITGBL1
***LMX1B***	LIM homeobox transcription factor 1 beta	p2@LMX1B
*MIR184*	MicroRNA 184	p1@MIR184
*MSMP*	Microseminoprotein, prostate associated	p1@MSMP, p3@MSMP
***PITX2***	Paired like homeodomain 2	p1@PITX2, p2@PITX2, p3@PITX2, p8@PITX2
***POU6F2***	POU class 6 homeobox 2	p3@POU6F2, p@chr7:39018373..39018384,+
*PTGDS*	Prostaglandin D2 synthase	p9@PTGDS, p10@PTGDS, p@chr9:139874657..139874682,−
*SHC4*	SHC adaptor protein 4	p3@SHC4, p5@SHC4
*SLC4A11*	Solute carrier family 4 member 11	p2@SLC4A11
*SLC4A4*	Solute carrier family 4 member 4	p7@SLC4A4
***TFAP2B***	Transcription factor AP-2 beta	p1@TFAP2B, p2@TFAP2B, p3@TFAP2B, p9@TFAP2B
*TSPAN6*	Tetraspanin 6	p4@TSPAN6
***ZFHX4***	Zinc finger homeobox 4	p6@ZFHX4

Transcription factors are shown in bold.

### 3.3 Expression of CEC-specific Promoters in the Human Eye

**Fig. 3:**
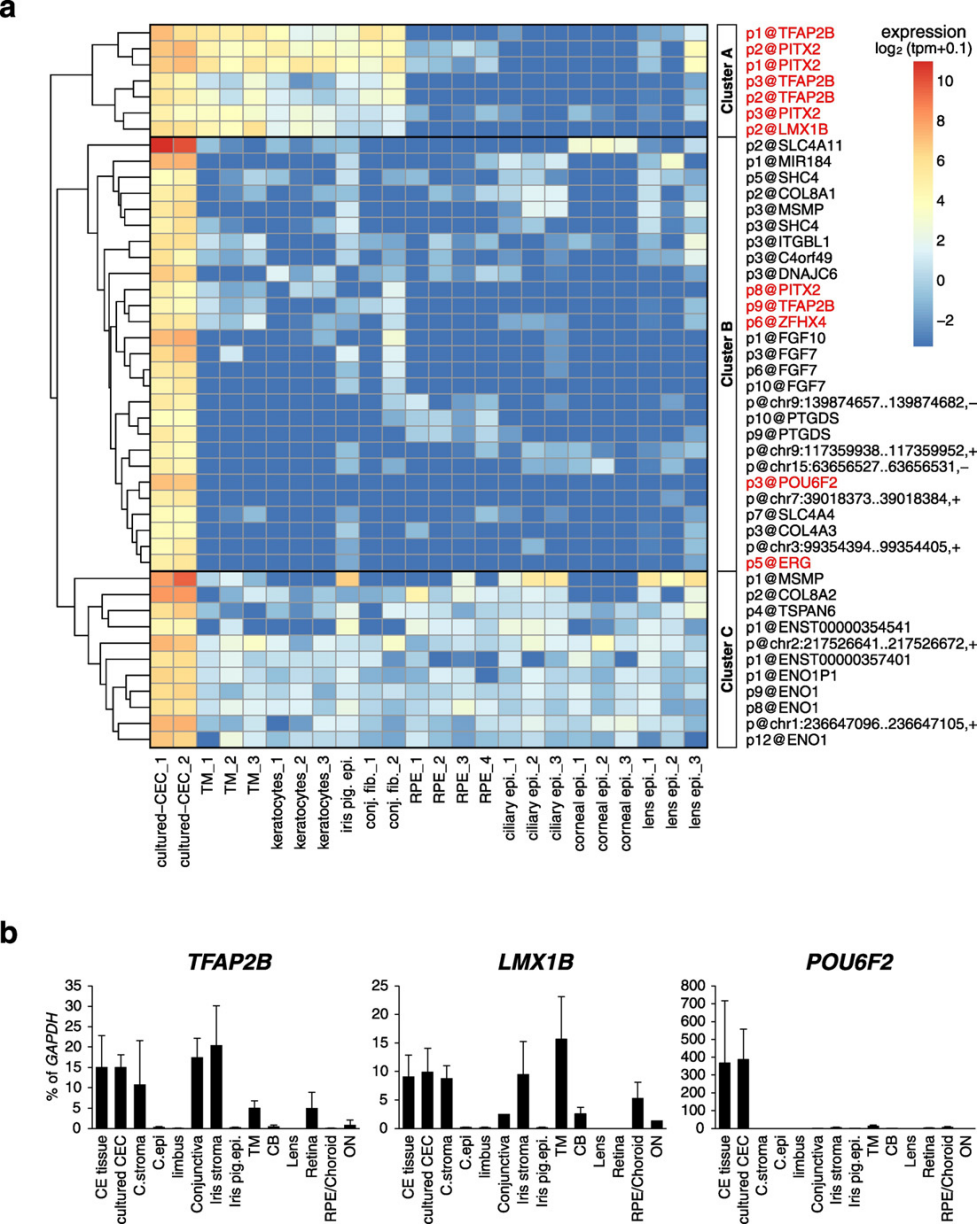
Expression of corneal endothelial cell-specific promoters in the human eye. (a) A heatmap of expression levels of 45 corneal endothelial cell (CEC)-specific promoters in human primary ocular cells. Hierarchical clustering analysis was performed using the complete linkage algorithm with the Euclidean distance matrix based on log_2_-scaled levels of promoter activity. Transcription factors are shown in red. TM: trabecular meshwork; iris pig. epi.: iris pigment *epi*thelial cells; conj. fib.: conjunctival fibroblast; RPE: retinal pigment epithelial cells; ciliary epi.: ciliary epithelial cells; corneal epi.: corneal epithelial cells; lens epi.: lens epithelial cells. (For interpretation of the references to color in this figure legend, the reader is referred to the web version of this article.) (b) qRT-PCR analysis of *TFAP2B*, *LMX1B*, and *POU6F2* expression in ocular tissues and cultured CECs. C. stroma: corneal stroma; C. epi.: corneal epithelial cells; iris pig. epi.: iris pigment epithelial cells; TM: trabecular meshwork; CB: ciliary body; RPE: retinal pigment epithelial cells; ON: optic nerve. Data are presented as the mean expression level (expressed in % of *GAPDH* expression level), and the error bars depict the standard deviation of four biological replicates.

Next, we examined the expression of the 45 CEC-specific promoters in the human eye. Because the FANTOM5 atlas includes transcriptome profiles of a wide range of ocular primary cells, we estimated the expression levels of these CEC-specific promoters across ocular primary cell samples and cultured CECs. Based on their expression pattern, we segregated the 45 CEC-specific promoters into three major clusters (Fig. 3a). Cluster A consisted only of transcription factors: three major promoters of *PITX2*, three major promoters of *TFAP2B*, and p2@LMX1B. These three transcription factors are highly expressed in trabecular meshwork cells and keratocytes as well as in CECs, *i.e.*, tissues that originate from neural crest-derived periocular mesenchyme. A recent study demonstrated that Pitx2 regulates Tfap2b expression, and Lmx1b expression was nominally decreased in *Pitx2*-knockout mice, although the latter did not reach statistical significance (Chen et al., 2016). Furthermore, it has been reported that Lmx1b is expressed in neural crest-derived cells, including CECs, during mouse eye development (Liu and Johnson, 2010). These reports, combined with our study, suggest that PITX2, TFAP2B, and LMX1B coordinate the differentiation of neural crest-derived periocular mesenchyme in the human eye. The promoters in cluster C are widely expressed at low levels in other ocular cells. Indeed, these promoters are highly expressed in CECs, however, their expression is not limited to CECs, and occurs broadly in different ocular cells. In contrast, cluster B consisted of promoters that exhibited a strictly CEC-specific expression pattern. p3@POU6F2 was included in this cluster, supporting the notion about CEC-specific expression of p3@POU6F2 in the human eye.

We used qRT-PCR to confirm expression patterns of these three CEC-specific transcription factors across ocular tissues and cultured CECs (Fig. 3b). As expected from the CAGE results, *LMX1B* and *TFAP2B* were highly expressed in neural crest-derived tissues, such as corneal stroma, trabecular meshwork, and iris stroma, as well as in CECs. Meanwhile, among all human ocular tissues examined, *POU6F2* expression was restricted to CE tissues and cultured CECs.

### 3.4 Dramatic Change in Gene Expression Profile During the Differentiation of HCEPs Towards CECs

**Fig. 4:**
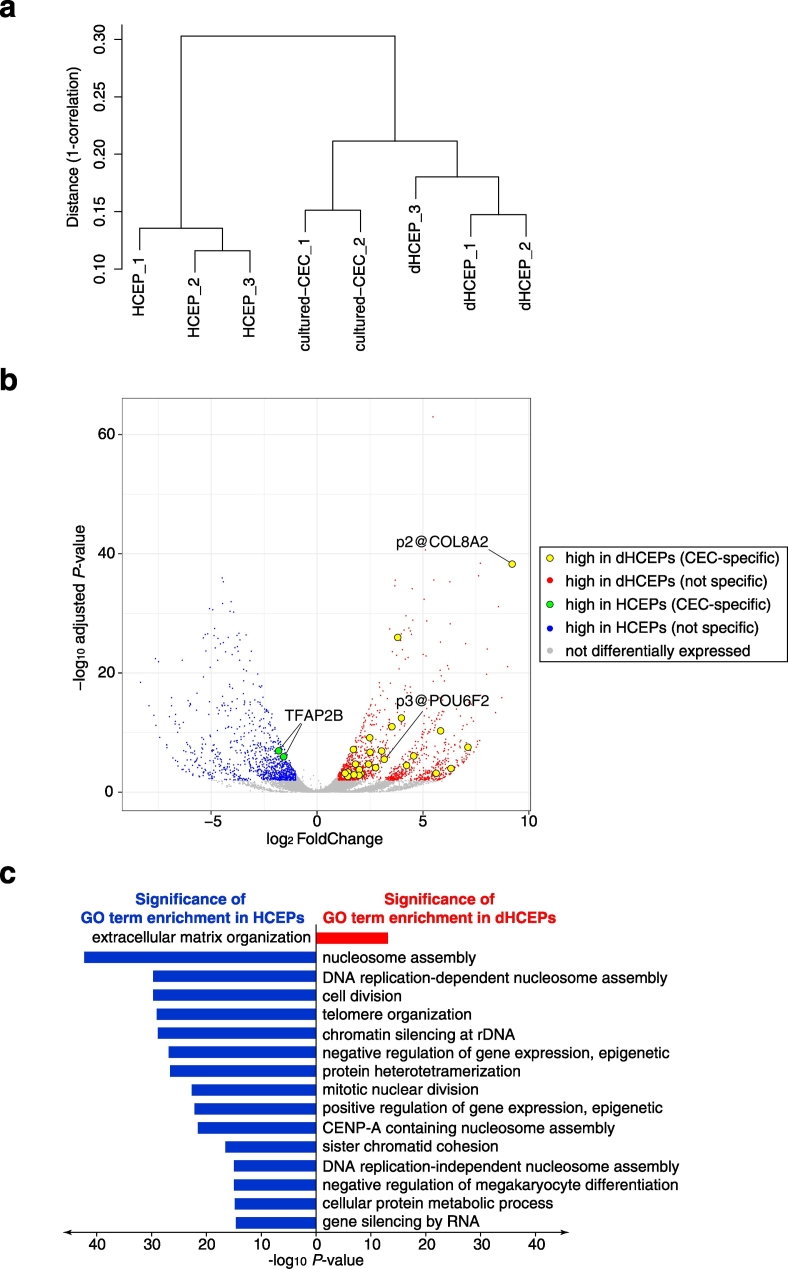
Transcriptome profiling of corneal endothelial progenitor cells and differentiated corneal endothelial progenitor cells. (a) Hierarchical clustering of corneal endothelial progenitor cell (HCEP), differentiated HCEP (dHCEP), and cultured corneal endothelial cell (CEC) samples based on the comprehensive analysis of their promoter activities by CAGE. Clustering analysis was performed using the average linkage algorithm with the Spearman correlation distance matrix. (b) Volcano plot of statistical significance against log_2_ fold-change in promoter expression levels between HCEPs and dHCEPs. Red dots represent promoters highly expressed in dHCEPs, and blue dots represent promoters highly expressed in HCEPs. Yellow dots represent CEC-specific promoters highly expressed in dHCEPs, and green dots represent CEC-specific promoters highly expressed in HCEPs. Promoters that were not differentially expressed between these two cell types are shown in gray. (For interpretation of the references to color in this figure legend, the reader is referred to the web version of this article.) (c) Gene ontology (GO) enrichment analysis of genes highly expressed in dHCEPs (red) and HCEPs (blue). GO terms with false discovery rate < 1 × 10^− 10^ are shown.

We next focused on the differentiation process of human CECs. We previously succeeded in isolating HCEPs and converting them into dHCEPs; these dHCEPs had pump function similar to that of CECs (Hara et al., 2014). First, we compared their gene expression profile with that of cultured CECs, to assess whether HCEPs properly differentiated into CECs. Hierarchical clustering analysis revealed that dHCEPs exhibited an expression pattern similar to that of cultured CECs, and this similarity was greater than that between HCEPs and dHCEPs derived from identical donors (Fig. 4a). This suggests that HCEPs become committed towards the CEC lineage upon their induction to dHCEPs.

Next, to explore transcriptional dynamics during CEC differentiation, we performed differential expression analysis between HCEPs and dHCEPs. During HCEP differentiation, 964 promoters were downregulated, and 1327 promoters were upregulated (Fig. 4b and Table S5a, b). GO enrichment analysis revealed that genes affected by downregulated promoters were enriched in cell proliferation-related terms, which was concordant with our previous report that revealed high proliferative capacity of HCEPs (Hara et al., 2014) (Table S6a and Fig. 4c, blue bars). In contrast, genes affected by upregulated promoters were enriched in extracellular matrix organization terms, including type IV and VIII collagen, which are major components of Descemet's membrane (Kabosova et al., 2007, Tamura et al., 1991) (Table S6b and Fig. 4c, a red bar). Because CECs produce collagens that form the Descemet's membrane throughout life (Bourne, 2003), these functional dHCEPs may contribute to collagen secretion.

Finally, we compared these differentially expressed promoters with the list of 45 CEC-specific promoters. Interestingly, only two CEC-specific promoters were downregulated during HCEP differentiation, and both of them were from the *TFAP2B* gene. Meanwhile, 25 of the 45 CEC-specific promoters (56%) were upregulated during HCEP differentiation (Fig. 4b). This observation also supports the notion that HCEPs properly differentiate towards CECs. The most differentially upregulated CEC-specific promoter was p2@COL8A2, which was also apparent in our previous study (Hara et al., 2014). Strikingly, p3@POU6F2 was significantly upregulated during HCEP differentiation. A previously reported RNA-seq dataset for human adult and fetal CECs (Chen et al., 2013) also demonstrated that *POU6F2* was significantly upregulated in adult CECs, whereas expression levels of genes encoding other CEC-specific transcription factors, such as *PITX2*, *LMX1B*, and *TFAP2B*, did not significantly change (Fig. S4). These results suggest that POU6F2 regulates terminal differentiation of CECs, whereas TFAP2B is mainly involved with the early stage of CEC development from neural crest cells.

### 3.5 CEC-specific Transcription Factor POU6F2 as a CEC Maturation Marker

**Fig. 5:**
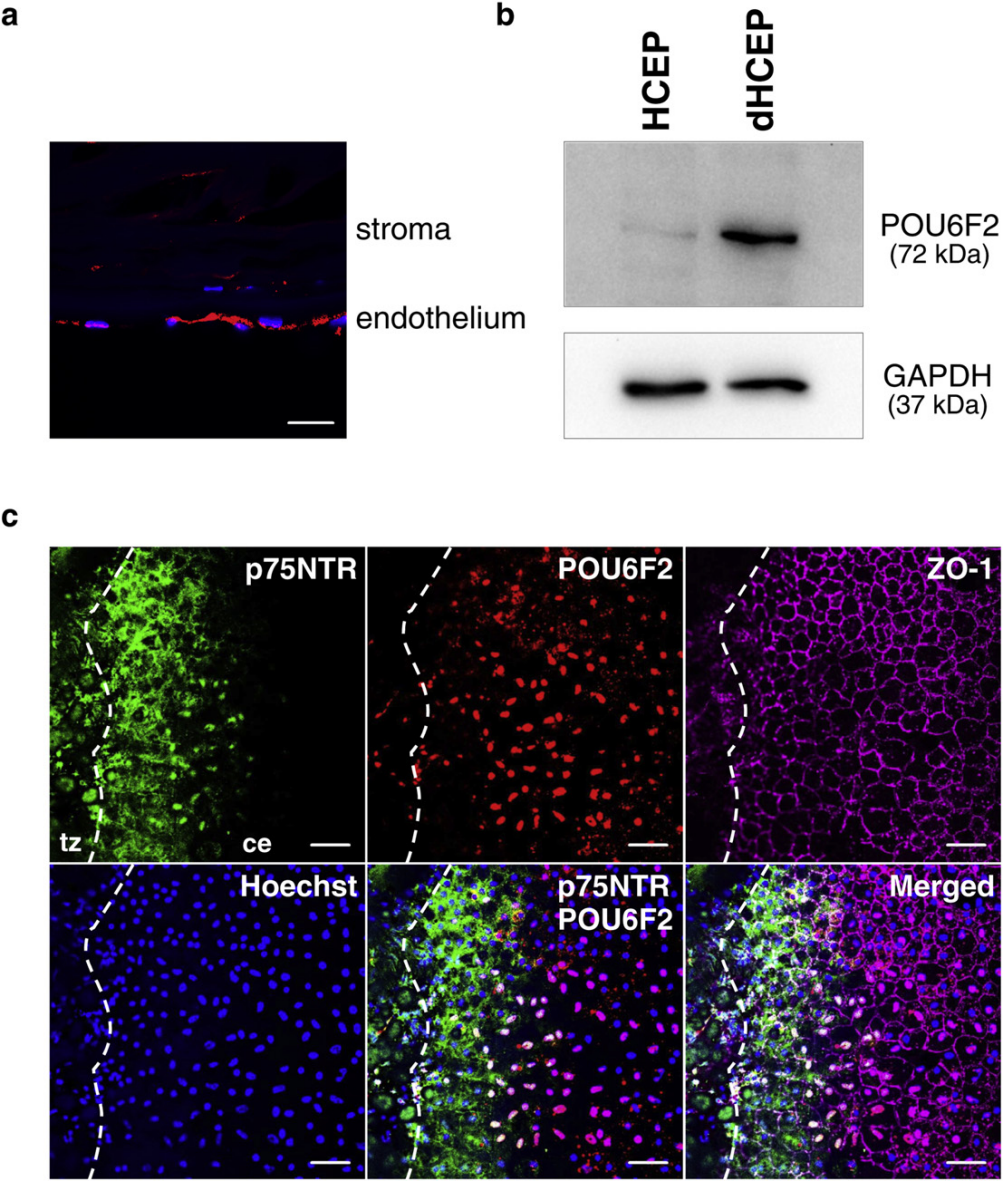
POU6F2 protein expression in corneal endothelial cells. (a) Immunofluorescence staining of human corneal tissue section. Red signals represent the expression of the POU6F2 protein detected by an anti-POU6F2 antibody. Hoechst 33342-stained nuclei are shown in blue. Scale bar: 20 μm. (For interpretation of the references to color in this figure legend, the reader is referred to the web version of this article.) (b) Western blotting analysis of corneal endothelial progenitor cells (HCEPs) and differentiated HCEPs (dHCEPs) using an anti-POU6F2 antibody. (c) Immunofluorescence staining of a flat-mounted human corneal tissue. Green signals represent the expression of the p75NTR protein, red signals represent the expression of the POU6F2 protein, and purple signals represent the expression of the ZO-1 protein at corneal endothelial cell (CEC) junctions. Hoechst 33342-stained nuclei are shown in blue. tz: transitional zone; ce: corneal endothelium. Dashed lines represent the edges between transitional zone and corneal endothelium. Scale bar: 50 μm.

These observations prompted us to examine the expression of POU6F2 in CECs at the protein level. It has been shown that POU6F2 exists mainly in two differentially spliced isoforms: one with a 36-amino acid insertion in the POU-specific subdomain, and the other without the insertion (Fig. S5a). As reported in human retina (Zhou et al., 1996) and brain (Fiorino et al., 2016), the isoform with the insertion was predominant in human CE tissues (Fig. S5b). To confirm the specific expression of POU6F2 protein in CECs, we performed immunofluorescence staining of human corneal tissue sections. The anti-POU6F2 antibody specifically stained corneal endothelium (Fig. 5a). Next, we confirmed the upregulation of the POU6F2 protein during CEC differentiation by western blotting analysis of HCEPs and dHCEPs (Fig. 5b). These results were consistent with our transcriptome analysis data.

Finally, we examined POU6F2 expression using flat-mounted human corneal tissue. It has been demonstrated that CECs located at the extreme periphery of human cornea express several stem cell and proliferation markers (He et al., 2012), suggesting that these CECs act as progenitors. In accordance with this finding, the expression of the p75NTR, a marker of neural crest cells, was enriched for CECs in the extreme periphery. Conversely, POU6F2 was not detected in these peripheral CECs and was mostly expressed in relatively central CECs that did not express p75NTR, indicating that POU6F2 is expressed in differentiated and mature CECs (Fig. 5c).

## 4 Discussion

To utilize stem cell-derived CECs for regenerative medicine, it is necessary to identify specific markers of CECs that enable quality assessment of final CEC products. We have previously reported several CEC-specific markers by comparing publicly available RNA-seq data with the FANTOM5 expression atlas (Yoshihara et al., 2015). However, we could not statistically assess their differential expression, because the protocols used were dissimilar. Those identified markers were indeed specifically expressed in CECs, but their expression levels were relatively low. In contrast, the design of the current study allowed us to compare promoter activities between CECs and other samples in the FANTOM5 atlas, because we performed CAGE analysis of CECs with the same protocol, using a HeliScope single molecule sequencer. After a careful quality check and elimination of inadequate data, we identified 137 and 206 promoters that were strictly specific to CE tissues and cultured CECs, respectively. Among them, 45 promoters at 28 gene loci were specifically expressed in both CE tissues and cultured CECs. It is noteworthy that several genes associated with corneal endothelial disorders were included in this set, which supports the notion that these promoters are crucial for CEC characteristics.

For differentiation from pluripotent cells or transdifferentiation, it is important to identify cell type-specific transcription factors that could be required to convert between cell types. We identified TFAP2B, LMX1B, and POU6F2 as CEC-specific transcription factors. Our qRT-PCR validation experiments were almost completely consistent with CAGE results, implying that our conclusion about the specificity of those transcription factors was valid. TFAP2B is a member of the transcription factor activating enhancer binding protein-2 (TFAP2, AP-2) family. It has been reported that mutations in *TFAP2B* cause Char syndrome, which is characterized by facial dysmorphism, patent ductus arteriosus, and finger anomalies (Satoda et al., 2000). Because these tissues derive from neural crest cells, TFAP2B has been suggested to regulate the migration or differentiation of neural crest cells. Recent studies demonstrated that deletion of *Tfap2b* in mice leads to the absence of corneal endothelium (Chen et al., 2016) or dysgenesis of multiple tissues in the anterior segment, which include the corneal endothelium, corneal stroma, ciliary body, and iridocorneal angle (Martino et al., 2016); these phenotypes are similar to those that occur due to *Pitx2*/*PITX2* disruption in mouse and human (Gage et al., 2014). LMX1B is a key regulator of the dorsoventral patterning of limbs, and mutations in *LMX1B* cause nail patella syndrome (Dreyer et al., 1998). This disorder affects not only the skeletal system, but also the kidneys and eyes (Sweeney et al., 2003). Morello et al. revealed that LMX1B directly regulates expression levels of type IV collagens COL4A3 and COL4A4, which are both required for glomerular basement membrane morphogenesis (Morello et al., 2001). Interestingly, we found that both COL4A3 and COL4A4 were specifically expressed in cultured CECs (Fig. S6), which strongly suggests that LMX1B regulates the expression of COL4A3 and COL4A4 in CECs. Although mutations in *LMX1B* have not been reported to cause human corneal disorders, there have been some reports that glaucoma is a comorbidity of nail patella syndrome (Lichter et al., 1997, Vollrath et al., 1998). Furthermore, *Lmx1b* mutant mice displayed dysgenesis of the corneal endothelium, corneal stroma, ciliary body, iris, and trabecular meshwork (Liu and Johnson, 2010, Pressman et al., 2000), which suggests that Lmx1b is an essential regulator of anterior segment morphogenesis. These observations, in addition to the specificity of PITX2, TFAP2B, and LMX1B expression to neural crest-derived ocular cells demonstrated in the present study, indicate that these transcription factors coordinately regulate the early development of the neural crest-derived periocular mesenchyme.

POU6F2 is a member of the POU family proteins which are involved in cell type-specific differentiation (Rosenfeld, 1991). However, there have been very few reports on POU6F2. POU6F2 was originally cloned from human retina, and is also known as retina-derived POU-domain factor-1 (RPF-1) (Zhou et al., 1996). POU6F2 has also been reported to be expressed in the developing midbrain (Zhou et al., 1996), pituitary (Yoshida et al., 2014), and kidneys (Di Renzo et al., 2006). However, our data demonstrated that the expression level of POU6F2 in CECs was considerably greater than in those tissues. Intriguingly, POU6F2 expression was reported to be decreased during neural and renal differentiation (Di Renzo et al., 2006, Yoshida et al., 2014), whereas we found that POU6F2 was upregulated during CEC differentiation. We further demonstrated that POU6F2 obtained from human CE tissues was mainly composed of the isoform with the 36-amino acid insertion (Fig. S5b). Although it has been shown that POU6F2 with the insertion lost its DNA-binding ability (Fiorino et al., 2016, Zhou et al., 1996), this isoform should have some biological function, because the insertion region is highly conserved in other species (Fig. S5a). Given that several genes related to corneal endothelial disorders are listed in CEC-specific genes, aberrations in POU6F2 might contribute to corneal endothelial disorders whose causative genes have not been identified.

To achieve the differentiation of CECs from stem cells *in vitro*, it is crucial to elucidate the molecular mechanisms of human CEC differentiation. Animal models are indeed useful for observing CEC development, however, there are some differences between human CECs and the CECs of other species. Importantly, human CECs have limited proliferative capability, unlike murine, rabbit, or bovine CECs (Joyce, 2005). We have previously succeeded in isolating HCEPs with high proliferative capability, and in the present study, we found that dHCEPs had a gene expression profile similar to that of cultured CECs. This suggests that HCEPs and dHCEPs represent a suitable *in vitro* model of human CEC differentiation. We further confirmed that a number of CEC-specific promoters were upregulated in dHCEPs. Interestingly, cell cycle-related genes were downregulated in dHCEPs, which is consistent with the fact that human CECs have limited proliferative capability even *in vitro*. Fiorino et al. showed that overexpression of POU6F2 reduced cell proliferation (Fiorino et al., 2016), which is consistent with our observation that POU6F2 is upregulated during HCEP differentiation. In contrast, TFAP2B was downregulated during the differentiation. These findings indicate that POU6F2 regulates terminal differentiation of CECs, whereas PITX2, TFAP2B, and LMX1B are required at the stage of periocular mesenchyme differentiation before commitment to differentiate into the CEC lineage.

In summary, we have comprehensively profiled promoter activities of human CECs, and identified 45 promoters at 28 gene loci that were specifically expressed in both CE tissues and cultured CECs. Among those loci, there were genes encoding three CEC-specific transcription factors: TFAP2B, LMX1B, and POU6F2. POU6F2 showed a particularly unique expression pattern. Of all the tissues studied, POU6F2 expression was restricted to CECs. Moreover, POU6F2 was upregulated during CEC maturation, indicating that it could be a marker of CEC differentiation or maturation. Thus, these transcription factors could be useful for the assessment and establishment of CECs derived from pluripotent stem cells in regenerative medicine.

The following are the supplementary data related to this article.

Table S1 Donor information, RNA integrity number (RIN), and overview of sequencing data for the CAGE analysis. COPD: chronic obstructive pulmonary disease; ESRD: end-stage renal disease. Table S1

Table S2 List of probes used for the Taqman Gene Expression assay and primers designed for the SYBR assay. Table S2

Table S3 (a) List of 137 promoters specifically expressed in corneal endothelial tissues. (b) List of 206 promoters specifically expressed in cultured corneal endothelial cells. Transcription factors are shown in bold. Promoters are sorted by statistical significance (adjusted *P*-value). Table S3

Table S4 Expression of the main promoter (p1) of transcription factors whose alternative promoters are specifically expressed in corneal endothelial cells. Top 10 samples are presented. Corneal endothelial cell (CEC) samples are shown in bold. CECs that do not belong to top 10 samples are masked with gray. p1@POU6F2 was not expressed in any primary cell samples or cultured CECs. N.D.: not detected. Table S4

Table S5 (a) List of 964 promoters highly expressed in corneal endothelial progenitors. (b) List of 1,327 promoters highly expressed in differentiated corneal endothelial progenitors. Corneal endothelial cell (CEC)-specific promoters are shown in bold. Promoters are sorted by statistical significance (adjusted *P*-value). Table S5

Table S6 (a) Gene ontology enrichment analysis of significantly upregulated genes in corneal endothelial progenitors. (b) Gene ontology enrichment analysis of significantly upregulated genes in differentiated corneal endothelial progenitors. GO terms with false discovery rate < 0.01 are shown. Table S6

Supplementary Figures S1–S6 Image 1

## Funding Sources

This work was supported by the Highway Program for Realization of Regenerative Medicine from the Japan Science and Technology Agency (JST), Japan Agency for Medical Research and Development (AMED) (KN), a MEXT Research Grant to the RIKEN CLST, and a MEXT Research Grant to the RIKEN Preventive Medicine and Diagnosis Innovation Program (YH). MY was supported by a RIKEN Junior Research Associate program and the Healthcare New Frontier Policy, Kanagawa Prefecture, Japan. These funding sources had no role in the study design, data collection, analysis, interpretation, or writing of this manuscript.

## Conflicts of Interest

The authors declare that they have no conflicts of interest.

## Author Contributions

MY prepared corneal tissue samples and CAGE libraries, conducted bioinformatics analyses and the literature search, and prepared figures and tables.
SH prepared cultured cell samples and conducted validation experiments.
SK provided tissue RNA samples.
MY and SH wrote the manuscript with assistance from MT and HK.
MY, SH, MT, MI, and HK designed the study.
YH and KN supervised the project.

All authors have read and approved the manuscript.
