# Supplementary material for: Restricted Presence of POU6F2 in Human Corneal Endothelial Cells Uncovered by Extension of the Promoter-level Expression Atlas
Source: eBioMedicine. 2017 Nov 4;25:175–86. doi: 10.1016/j.ebiom.2017.10.024 (PMC5704063; doi:10.1016/j.ebiom.2017.10.024)
Supplement: Supplementary file 7 — Supplementary Figures S1–S6 [file mmc7.pdf]

Supplementary Data

Restricted presence of POU6F2 in human corneal endothelial cells uncovered by extension of the promoter-level expression atlas

Masahito Yoshihara, Susumu Hara, Motokazu Tsujikawa, Satoshi Kawasaki, Yoshihide Hayashizaki, Masayoshi Itoh, Hideya Kawaji, Kohji Nishida

a

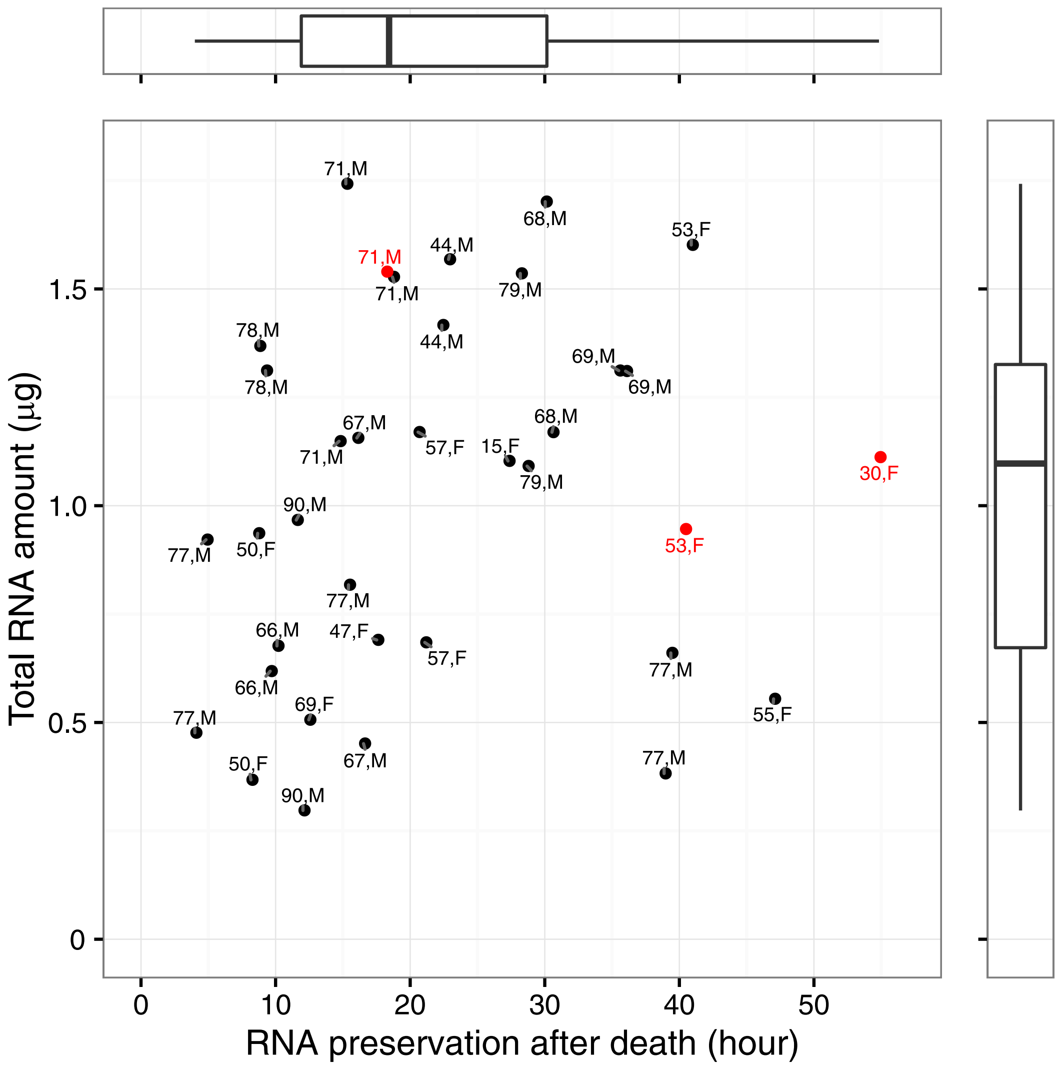

b

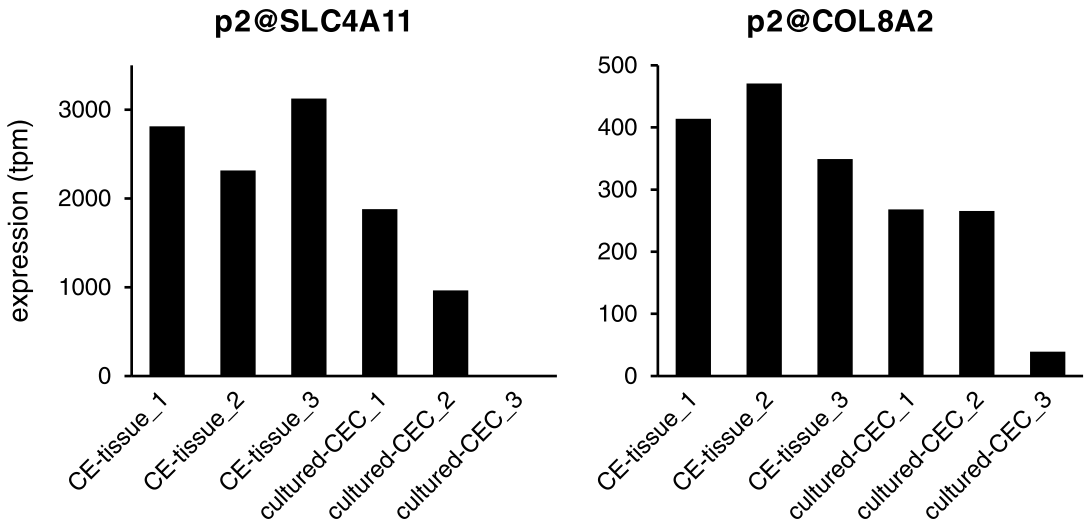

Fig. S1. Corneal endothelial tissue sampling and quality check of the CAGE data.

(a) Total amount of extracted RNA and RNA preservation time after death of 36 fresh corneal endothelial tissue samples. Each dot represents an individual corneal endothelial tissue sample. Donor age and sex are shown (M: Male, F: Female). The three corneal endothelial tissue samples used in this study are shown in red.
(b) Expression levels of well-known corneal endothelial cell-markers in corneal endothelial cell samples analyzed by CAGE. Expression levels of p2@SLC4A11 and p2@COL8A2 are shown.

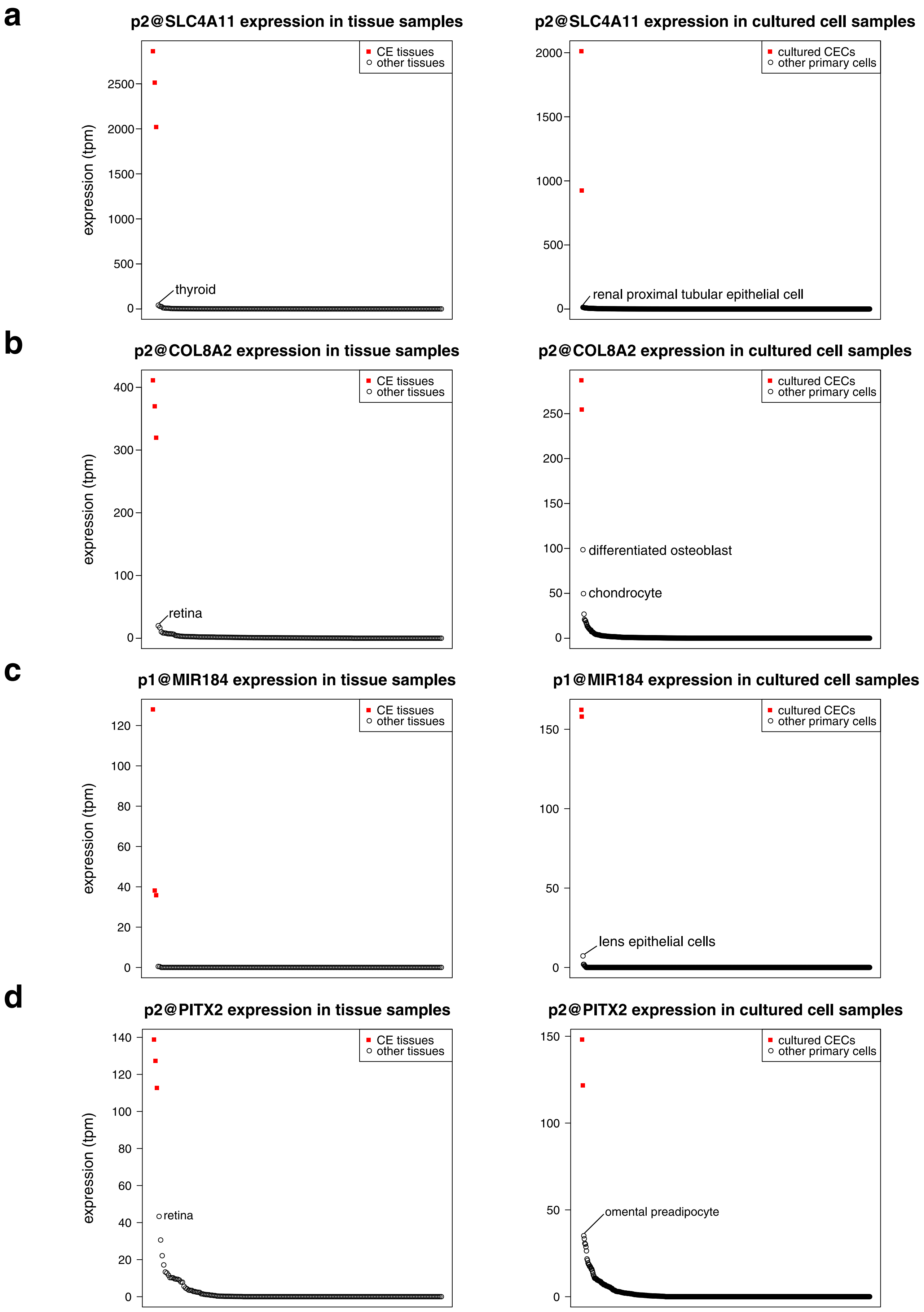

Fig. S2. Expression levels of corneal endothelial disorder-related genes in corneal endothelial cells and various human tissues or cells.

Expression levels of p2@SLC4A11 (a), p2@COL8A2 (b), p1@MIR184 (c), and p2@PITX2 (d) are shown. Red squares represent expression levels in corneal endothelial cells (CECs), and black circles represent expression levels in other tissues or cells. Samples are sorted from left to right in the order of the expression level of each promoter. The y-axes represent expression levels (tpm).

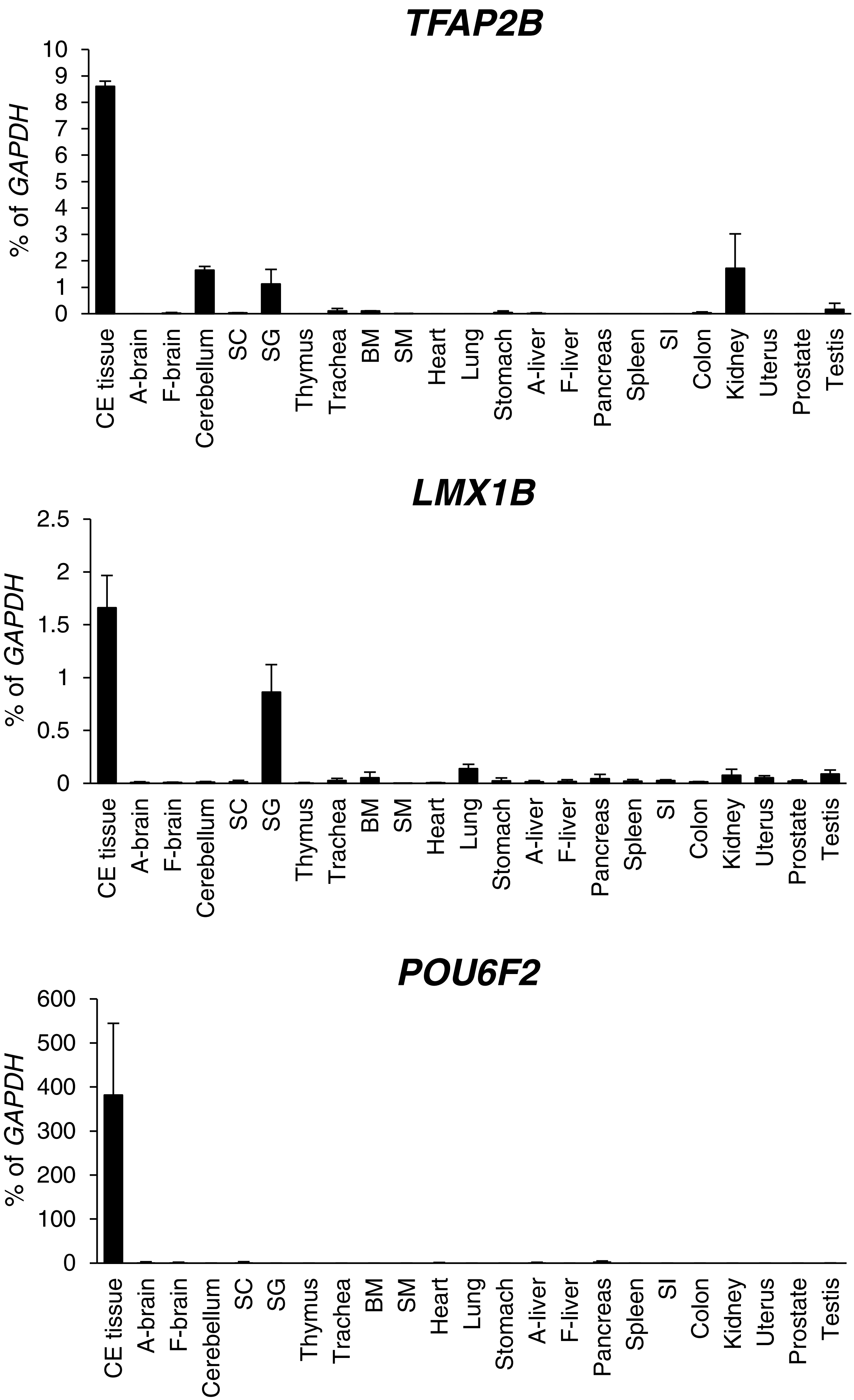

Fig. S3. qRT-PCR analysis of *TFAP2B*, *LMX1B*, and *POU6F2* expression in the human body.

A-brain: adult brain; F-brain: fetal brain; SC: spinal cord; SG: salivary gland; BM: bone marrow; SM: skeletal muscle; A-liver: adult liver; F-liver: fetal liver; SI: small intestine. Data are presented as the mean expression level (expressed in % of *GAPDH* expression level), and the error bars depict the standard deviation of technical duplicates.

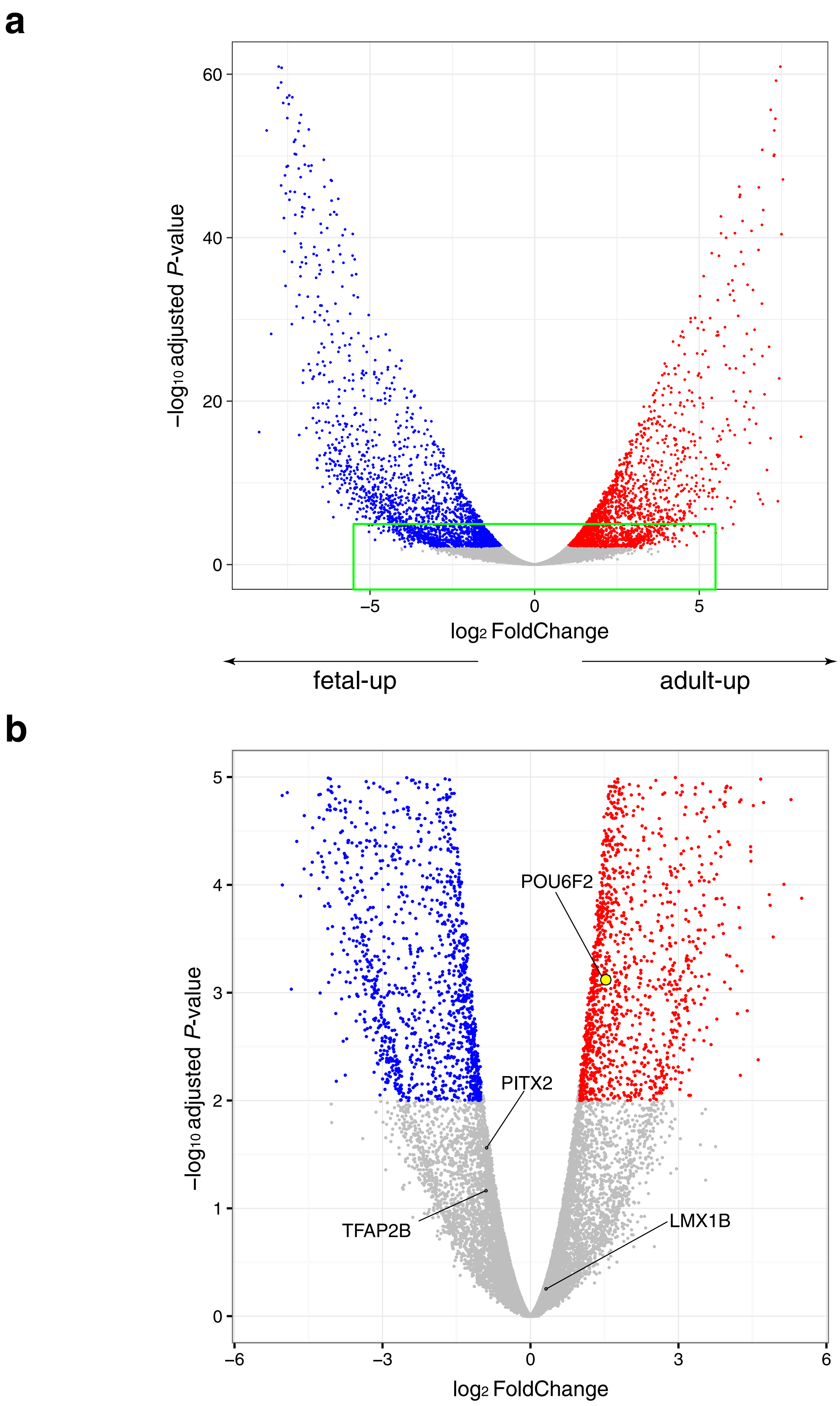

Fig. S4. Volcano plot of statistical significance against log2 fold-change in gene expression levels between adult and fetal corneal endothelial cells.

RNA-seq data were obtained from GSE41616. Red dots represent genes highly expressed in adult corneal endothelial cells (CECs), and blue dots represent genes highly expressed in fetal CECs. Genes that were not differentially expressed between these two groups of CECs are shown in gray. (b) shows a magnified view of the region indicated by a green rectangle in (a).

a

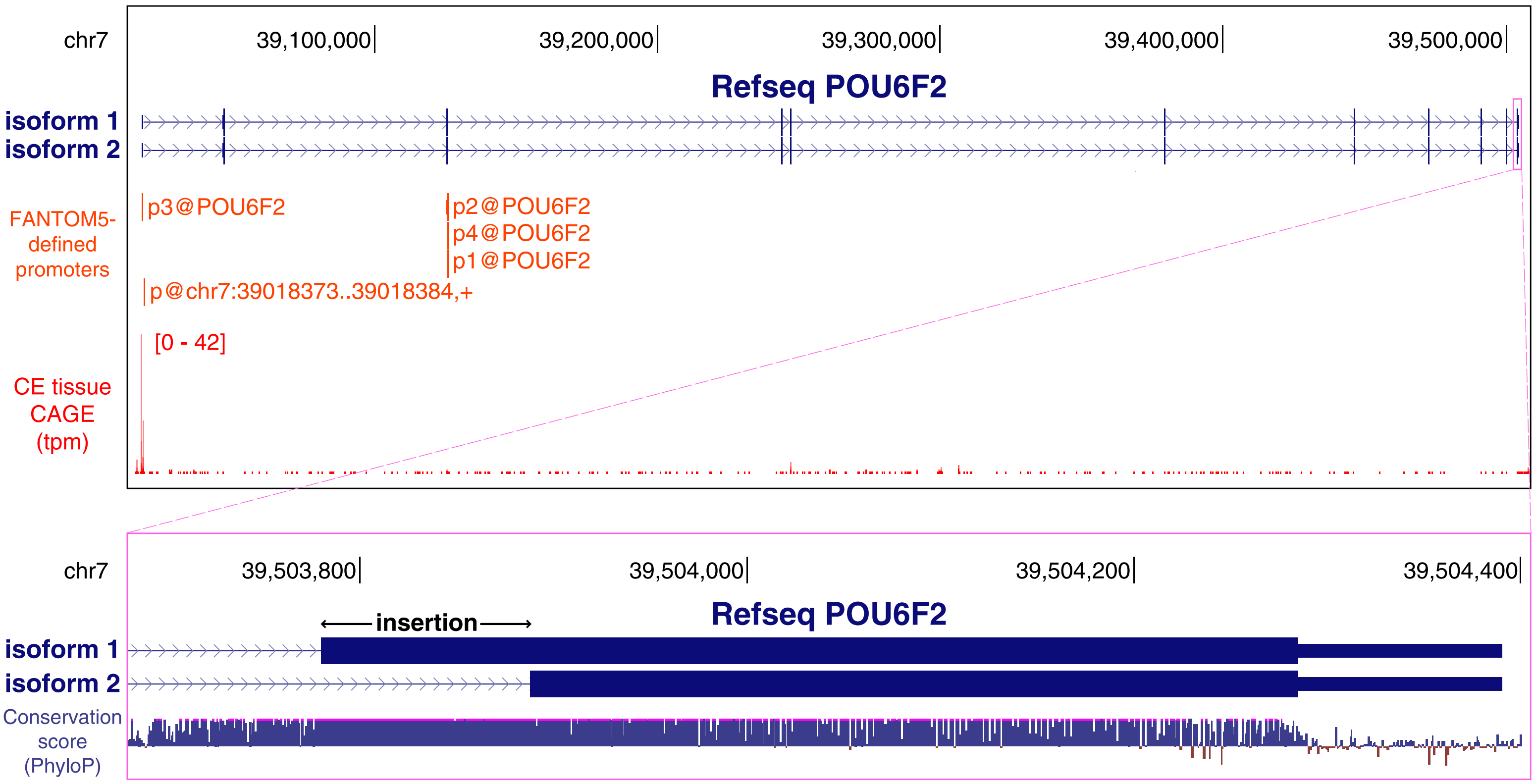

b

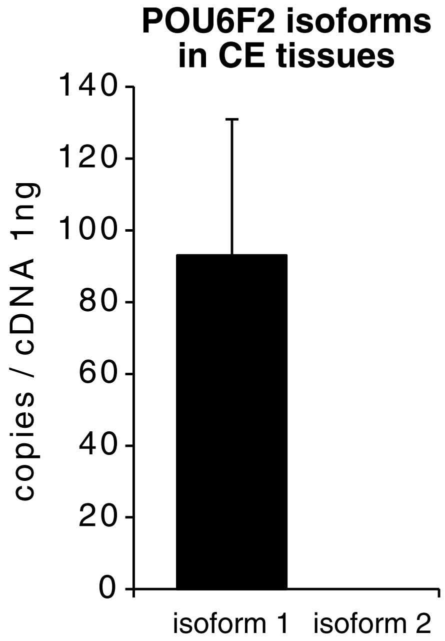

Fig. S5. Full structure of the *POU6F2* gene and its two major isoforms.

(a) Genome browser view of *POU6F2*. (Top) Full structure of *POU6F2*. CAGE peaks are observed at p3@POU6F2 in CE tissue samples. p1, p2, and p4 promoters are identified around exon 3 in the FANTOM5, however, signal peaks cannot be observed at these promoter regions in CE tissue samples. (Bottom) Zoomed-in view of exon 11. There is a 36-amino acid (108 base pair) insertion in isoform 1, and this insertion region is highly conserved across species. **(b)** Expression levels of isoform 1 and isoform 2 of *POU6F2* in CE tissue samples, quantified by qRT-PCR. The y-axes represent the number of copies per 1 ng cDNA, and the error bar depicts the standard deviation of four biological replicates.

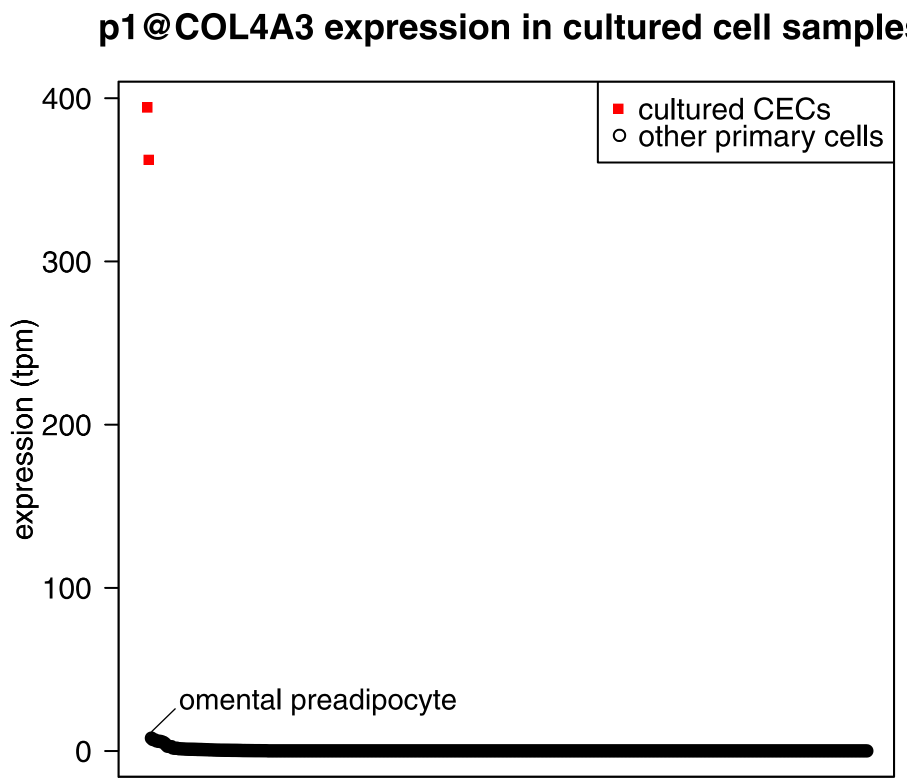

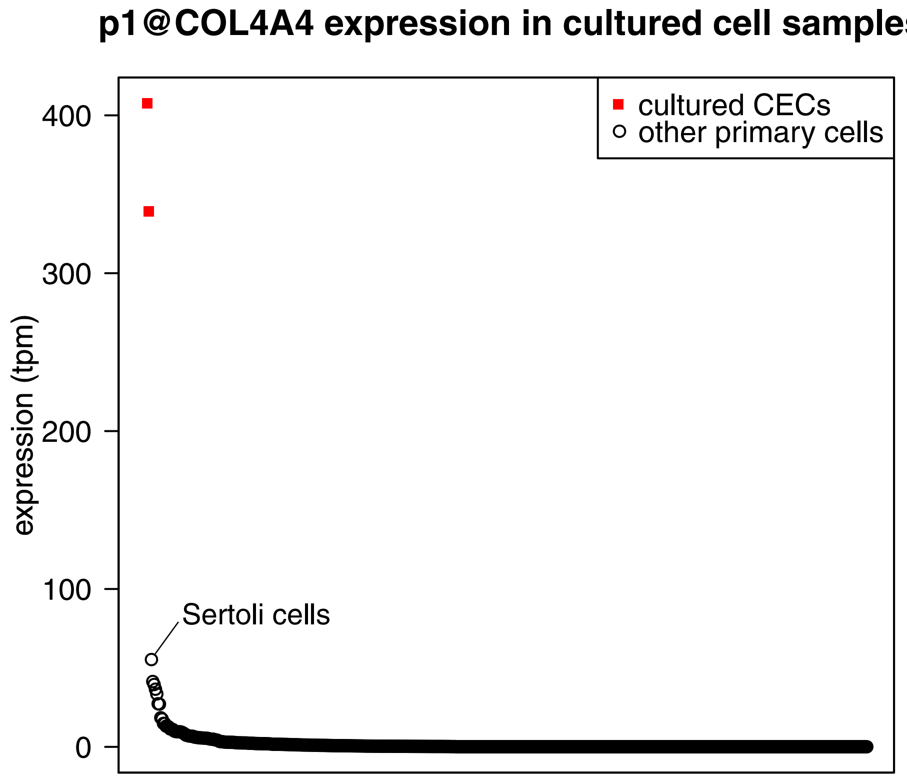

Fig. S6. Expression levels of p1@COL4A3 and p1@COL4A4 in cultured corneal endothelial cells and various human primary cells.

Red squares represent expression levels in cultured corneal endothelial cells (CECs), and black circles represent expression levels in other cells. Samples are sorted from left to right in the order of the expression level of each promoter. The y-axes represent expression levels (tpm).
